# Associations between consumption of three types of beverages and risk of cardiometabolic multimorbidity in UK Biobank participants: a prospective cohort study

**DOI:** 10.1186/s12916-022-02456-4

**Published:** 2022-08-18

**Authors:** Yi Luo, Lingfang He, Tianqi Ma, Jinchen Li, Yongping Bai, Xunjie Cheng, Guogang Zhang

**Affiliations:** 1 Department of Cardiovascular Medicine, The Third Xiangya Hospital, Central South University, 138 Tongzipo Road, Changsha, 410013 Hunan China; 2 Department of Geriatric Medicine, Center of Coronary Circulation, Xiangya Hospital, Central South University, 87 Xiangya Road, Changsha, 410008 China; 3 National Clinical Research Center for Geriatric Disorders, Xiangya Hospital, Central South University, Changsha, 410008 China; 4 Department of Cardiovascular Medicine, Xiangya Hospital, Central South University, Changsha, 410008 China

**Keywords:** Beverages, Cardiometabolic multimorbidity, Sugar-sweetened beverages, Artificially sweetened beverages, Pure fruit/vegetable juices

## Abstract

**Background:**

Although the association between beverages and a single cardiometabolic disease has been well studied, their role in disease progression from the single cardiometabolic disease state to cardiometabolic multimorbidity (CMM) state remains unclear. This study examined the associations between three types of beverages: sugar-sweetened beverages (SSBs), artificially sweetened beverages (ASBs), and pure fruit/vegetable juices, and the incidence of CMM in patients with a single cardiometabolic disease.

**Methods:**

Our analysis included 37,994 participants from the UK Biobank who completed at least one dietary questionnaire and were diagnosed with only one cardiometabolic disease at the time of recruitment. Competing risk models were used to examine the association between the three types of beverages and incidence of CMM. We conducted analysis both in patients with any single cardiometabolic disease and in patients with specific cardiometabolic disease.

**Results:**

During a median follow-up of 9.1 years (interquartile range [IQR] 9.0–9.8), a total of 6399 participants developed CMM. The consumption of SSBs and ASBs (>1 serving per day) was associated with a higher risk of CMM (SSBs: hazard ratio [HR] 1.19, 95% confidence interval [95% CI] 1.08–1.31; ASBs: HR 1.15, 95% CI 1.04–1.27). Intake of pure fruit/vegetable juices was inversely associated with the incidence of CMM (0–1 serving per day: HR 0.90, 95% CI 0.85–0.94; >1 serving per day: HR 0.90, 95% CI 0.81–0.99). However, the association of the high-level consumption of pure fruit/vegetable juices (>1 serving per day) was not statistically significant after correcting for multiple testing. In the analysis of patients with specific cardiometabolic diseases, positive associations were observed in patients with hypertension for SSBs consumption, while inverse associations persisted in patients with cardiovascular disease (coronary heart disease or stroke) and in hypertensive patients for pure fruit/vegetable juice consumption.

**Conclusions:**

Consuming >1 serving of SSBs and ASBs per day was associated with a higher risk of CMM in patients with a single cardiometabolic disease. In contrast, intake of pure fruit/vegetable juices was inversely associated with the risk of CMM. Our findings highlight the need to limit the use of SSBs and ASBs in patients with a single cardiometabolic disease.

**Supplementary Information:**

The online version contains supplementary material available at 10.1186/s12916-022-02456-4.

## Background

Multimorbidity, which refers to the co-occurrence of two or more long-term conditions in the same individual, is emerging as an international healthcare challenge and a global health research priority [1]. Cardiometabolic diseases, such as diabetes, hypertension, coronary heart disease, and stroke, are prevalent chronic conditions with relatively similar aetiologies and the leading cause of health burden and mortality worldwide [2]. Owing to advances in health care, survival after the onset of the first cardiometabolic disease has been prolonged. However, the likelihood of developing another is higher, causing cardiometabolic multimorbidity (CMM) to be very common in these patients [3]. A study in Canada revealed that 32.2% of patients with heart disease, 22% of patients with diabetes, and 48.4% of stroke survivors reported having another cardiometabolic disease [4]. Multimorbidity has been reported to be associated with a lower quality of life [5], higher health care use [6], more complications [7], and a greater risk of disability [8] and mortality [9–11]. A recent study showed that each additional cardiometabolic disease may double the risk of mortality [11]. Previous studies have shown that lifestyle behaviours, including smoking and alcohol consumption, are associated with a higher risk of CMM; however, little is known about the role of dietary factors, especially sweet beverages, in disease progression from the single cardiometabolic disease state to CMM state [12–15].

The adverse effects of sugar-sweetened beverages (SSBs), including carbonated and noncarbonated soft drinks, fruit drinks, and sports drinks, on cardiometabolic health have long been a matter of much public concern [16, 17]. A robust body of evidence has demonstrated that SSBs intake is associated with a higher risk of obesity [18], hypertension [19–21], type 2 diabetes [21–23], cardiovascular disease [24, 25], and other adverse health outcomes [26–28]. Several beverages are perceived as healthy alternatives to SSBs, including artificially sweetened beverages (ASBs) and pure fruit/vegetable juices. However, previous studies have found conflicting impacts of these alternatives on cardiometabolic health; some reported harmful effects of ASBs and fruit juice consumption on cardiometabolic diseases [21, 22, 24, 29, 30], while others observed inverse or non-significant associations with health outcomes [19, 31–34]. Most epidemiological studies which investigated the association of these three types of beverages with cardiometabolic diseases were conducted in healthy individuals. However, little is known about their effect on CMM risk in individuals with a single cardiometabolic disease. It is inappropriate to directly assume the same implications of these three types of beverages in the incidence of CMM since evidence suggests that one risk factor may exhibit distinct effects on different processes in the course of cardiometabolic diseases, such as from healthy state to the single cardiometabolic disease state or from the single cardiometabolic disease state to CMM [12, 13]. Previous studies have shown contradictory results in the association between ASBs and pure fruit/vegetable juice consumption and the risk of cardiometabolic diseases in healthy individuals, and it is unclear whether these associations apply to individuals with a single cardiometabolic disease.

This study aimed to investigate the associations between consumption of three types of beverages (SSBs, ASBs, and pure fruit/vegetable juices) and the risk of CMM in patients with a single cardiometabolic disease. Our study aimed to show the role of these three types of beverages in the secondary prevention of cardiometabolic diseases and provide more evidence for restricting their consumption.

## Methods

### Study population

The UK Biobank is a prospective population-based cohort study that recruited 502,414 participants aged 40–69 years at baseline. Between March 2006 and July 2010, participants were invited to one of 22 assessment centres across the UK. The participants were mostly white Europeans (94.1%), with a small number of Asians (2.3%), blacks (1.6%), and people of other ethnic origins. They also provided detailed baseline information on sociodemographic factors, lifestyle behaviours, and health-related history via a self-completed touchscreen questionnaire and computer-assisted interview. Anthropometric measurements and biological samples were obtained as well. The UK Biobank has full ethical approval from the National Health Service (NHS) National Research Ethics Service. All individuals voluntarily participated in the study and provided informed written consent for participation and follow-up.

### Inclusion and exclusion criteria

Of the 502,414 UK Biobank participants, 210,970 completed the online 24-h dietary recall questionnaire on at least one occasion. Of these, 47,472 participants had a history of a single cardiometabolic disease (coronary heart disease, hypertension, stroke, or diabetes) at baseline and were included in the analysis. Participants who had missing data (*n* = 8830; *n* = 7905 due to missing data on physical activity) or who reported dubious intake of total energy (*n* = 648; < 500 or >3500 kcal/day for women, <800 or >4200 kcal/day for men) were excluded, as in a previous study [26], leaving 37,994 participants for analysis (Additional file 1: Fig. S1).

### Assessment of beverage consumption

Dietary information was collected using a web-based dietary assessment tool (Oxford WebQ), which is based on a set of elaborate questions on the intake of up to 206 types of foods and 32 types of beverages consumed during the previous 24 h. The intake of beverages was assessed by asking participants how many glasses/cans/cartons/250 mL SSBs (carbonated drinks, fruit drinks, squash and cordial), ASBs (low-calorie and diet drinks), and pure fruit/vegetable juices they had consumed the previous day. The Oxford WebQ has been validated against a traditional interviewer-based 24-h dietary recall, as a suitable method for measuring dietary intake in large population studies [35, 36]. Participants were invited to complete the Oxford WebQ at baseline, with four separate follow-up occasions between February 2011 and June 2012. Of the 37,994 participants in our analysis, 39.9% completed the questionnaire once, 22.6% twice, 20.2% three times, 14.5% four times, and 2.8% five times. We used the mean dietary consumption of participants who completed more than one questionnaire in our main analysis and the consumption from the baseline questionnaire in the sensitivity analysis. The Pearson correlation coefficient between mean beverage consumption and beverage consumption from the baseline questionnaire was 0.874 for SSBs, 0.898 for ASBs, and 0.876 for pure fruit/vegetable juices.

### Assessment of covariates

Information on sociodemographic and lifestyle behaviours were ascertained at baseline using a self-reported online questionnaire, which included age, sex, ethnicity, alcohol consumption, food and vegetable consumption, red meat consumption, sedentary behaviour (using computers, driving, and watching television), smoking status, and drug use (insulin, antihypertensive drugs, lipid-lowering drugs, and aspirin). The area-based Townsend deprivation index was used to assess socioeconomic status, which was derived from consensus data on employment, housing, car ownership, and household overcrowding, corresponding to the postcode of residence. Physical activity was assessed at recruitment using a questionnaire based on the International Physical Activity Questionnaire (IPAQ), regarding the duration and frequency of different-intensity activities. The overall energy expenditure from physical activity was derived from the summed metabolic equivalents (MET-h/week) which were calculated by multiplying the duration of light, moderate, and vigorous physical activity per week by the weights of 2.5, 4, and 8, respectively, and then summing them. Body mass index (BMI) was calculated by dividing the weight (kg) by height squared (m^2^). The total sugar, fat, and energy intake for each participant were provided by the UK Biobank, which was calculated by multiplying the quantity consumed by the nutrient composition of the food or beverage, as taken from the UK food composition database, McCance and Widdowson’s The Composition of Foods and its supplements [37]. More detailed information on all covariates is provided in Additional file 2: Table S1.

### Ascertainment of outcomes

The main outcome was CMM, defined as progression to at least two of the following cardiometabolic diseases: coronary heart disease, hypertension, stroke, and diabetes. Participants were regarded as having cardiometabolic diseases if they had either a self-reported diagnosis, cardiometabolic disease medication, or surgery history on the touchscreen questionnaire or by verbal interview or via electronic health records which were consistent with the diagnosis of cardiometabolic disease. The electronic in-patient data was accessed by linkage to the National Health Service (NHS) Digital for England, Information and Statistics Division for Scotland, and Secure Anonymised Information Linkage for Scotland. The specific diagnostic criteria according to a previous study are shown in Additional file 3: Table S3 [38]. For each participant with the cardiometabolic disease, the date of diagnosis was compared with the date of recruitment to determine whether the onset occurred before recruitment or at follow-up. The date of onset of CMM was considered the earliest date of the second cardiometabolic disease during the follow-up period, ascertained via any of the data sources. Deaths were ascertained via linkage to the death register data from NHS Digital for England and Wales and the NHS Central Register, National Records of Scotland for Scotland.

### Study design and statistical analyses

In this population-based prospective cohort study, participants were categorised into three groups, based on their consumption of SSBs, ASBs, and pure fruit/vegetable juices as follows: 0, 0–1, >1 servings per day, respectively. The baseline characteristics of the participants are expressed as mean (standard deviation) or number (percentage) and compared among different beverage consumption groups using Student’s *t*-tests and chi-square tests for continuous and categorical variables, respectively. The Fine and Gray competing risk model was used to estimate the cumulative incidence of CMM and death. We also calculated hazard ratios (HRs) and 95% confidence intervals (95% CI) of CMM risks for beverage consumption in patients with a single cardiometabolic disease using the multivariable Fine and Gray competing risk model. The follow-up period began at the time when the participants completed their last 24-h dietary questionnaire and continued until the first occurrence of CMM, death, or the end of study period (June 2021), whichever came first. Linear trends were tested using the median intake, representing each category as a continuous variable in the multivariable competing risk model. To assess potential differences in the associations between beverage consumption and CMM risk in patients with specific cardiometabolic diseases, we conducted the analyses in patients with hypertension, patients with diabetes, and patients with cardiovascular disease (stroke or coronary heart disease), respectively. Participants who consumed 0–1 serving per day and participants who consumed >1 serving per day were combined (>0 serving per day) in the analysis for cardiovascular disease and diabetes due to the small sample size. Four multivariable-adjusted models were fitted in the analyses for patients with any single cardiometabolic disease: model 0 was adjusted for sociodemographic factors, including age, sex, ethnicity, and deprivation index; model 1 was additionally adjusted for BMI and lifestyle behaviours, including smoking status (current smokers or non-current smokers), alcohol consumption (over three times a week or not), physical activity, and sedentary time; model 2 was additionally adjusted for dietary factors, including total sugar, energy, fat, vegetable and fruit intake, fish intake, and red meat intake; and model 3 was additionally adjusted for drug use, including insulin, antihypertensive drugs, lipid-lowering drugs, and aspirin use. Model 3 was fitted in the analysis for patients with specific cardiometabolic diseases.

Eight sensitivity analyses were conducted to test the robustness of our results. First, we eliminated the first 2 years of follow-up to minimize reverse causality. Second, we recalculated the risks after excluding participants who reported losing weight compared with 1 year before recruitment in a baseline touchscreen questionnaire to decrease the effects of going on a diet. Third, we re-ran the model using beverage intake ascertained only from the baseline questionnaire instead of the mean intake of all questionnaires completed to approach baseline status. Fourth, to test for residual confounding of alcohol consumption, we re-ran the models by replacing alcohol consumption frequency (drinking ≥ 3 times per week or not) with alcohol consumption units (≥14 units per week or not). Fifth, we recalculated the models using a different approach to assess the physical activity. We divided the participants into two groups based on whether they met the 2017 UK Physical Activity Guidelines of 150 min of walking or moderate activity per week or 75 min of vigorous activity. Sixth, to investigate whether the association of beverage consumption were partially mediated by obesity, we re-ran the models without adjusting for BMI. Seventh, to minimize the confounding effects of other beverage consumption, we re-ran the analysis mutually adjusted for three different types of beverages, that is, SSBs were adjusted for ASBs and pure fruit/vegetable juice intake, and vice versa. We also conducted a stratified analysis with several covariates selected prior to data analysis as in previous study, such as age, sex, ethnicity, deprivation index, smoking status, alcohol consumption, physical activity, sedentary time, and BMI [33]. Lastly, we used a multi-state model (MSM) to recalculate the associations between the three types of beverages and cardiometabolic outcomes, which is a classical and reliable approach for multimorbidity studies. The MSM approach allowed for simultaneous estimation of the role of risk factors in the transitions from healthy to single cardiometabolic disease and from single cardiometabolic disease to CMM [39, 40]. Two-tailed *P* < 0.05 was considered statistically significant. The Benjamini-Hochberg correction was used for *P*-values in the main analysis and *P* for interaction in the stratified analysis to account for multiple comparisons. All statistical analyses were performed using R software (version 4.1.0).

## Results

Of the 37,994 participants with 331,645 person-years of follow-up (median per participant of 9.1 years, IQR 9.0–9.8 years), we ascertained 6399 incident cases of CMM. Among all participants in our analysis, 12,479 (32.8%) drank SSBs, of whom 9868 (26.0%) had ≤1 serving per day and 2611 (6.9%) had >1 serving per day; 8493 (22.4%) drank ASBs, of whom 6194 had (16.3%) ≤1 serving per day and 2299 (6.1%) had >1 serving per day; 19,580 (51.5%) drank pure fruit/vegetable juices, of whom 16,876 (44.4%) had ≤1 serving per day and 2704 (7.1%) had >1 serving per day. The baseline characteristics of participants according to SSBs, ASBs, and pure fruit/vegetable juice consumption are shown in Table 1. Participants with higher consumption of both SSBs and ASBs were more likely to be younger, had a lower socioeconomic status, and spent more sedentary time. Although higher SSBs and ASBs consumers both had higher BMI and consumed fewer vegetables and fruits, higher SSBs consumers had higher daily total energy, sugar, and fat intake, in contrast to consumers with higher ASBs. Meanwhile, consumers of pure fruit/vegetable juices had a lower BMI and consumed more vegetables and fruits despite higher daily total energy, sugar, and fat intake (all *P* < 0.01)

**Table 1:** Baseline characteristics of participants according to three types of beverage consumption in UK Biobank at 2021 (*N*=37,994)*

	Sugar-sweetened beverages			Artificially sweetened beverages			Pure fruit/vegetable juices		
	0/day	0–1/day	>1/day	0/day	0–1/day	>1/day	0/day	0–1/day	>1/day
Number of participants (*n*)	25,515	9868	2611	29,501	6194	2299	18,414	16,876	2704
Male %	12,772 (50.1)	5291 (53.6)	1570 (60.1)	15,614 (52.9)	2960 (47.8)	1059 (46.1)	9033 (49.1)	9012 (53.4)	1588 (58.7)
White ethnicity %	24,542 (96.2)	9398 (95.2)	2441 (93.5)	28,219 (95.7)	5944 (96.0)	2218 (96.5)	17,560 (95.4)	16,301 (96.6)	2520 (93.2)
Age (years)	61.6 (7.1)	61.3 (7.4)	58.9 (7.9)	61.8 (7.1)	60.3 (7.5)	58.3 (7.8)	60.8 (7.4)	62.0 (7.1)	61.2 (7.3)
Deprivation index^a^	−1.6 (2.9)	−1.6 (2.9)	−1.3 (3.0)	−1.6 (2.9)	−1.6 (2.8)	−1.3 (3.1)	−1.4 (2.9)	−1.8 (2.8)	−1.4 (3.0)
Sedentary hours (h/day)	4.9 (2.3)	5.1 (2.4)	5.5 (2.6)	4.9 (2.4)	5.3 (2.4)	5.6 (2.5)	5.2 (2.5)	4.9 (2.3)	4.9 (2.4)
Current smokers %	1775 (7.0)	632 (6.4)	222 (8.5)	2040 (6.9)	418 (6.7)	171 (7.4)	1537 (8.3)	918 (5.4)	174 (6.4)
Drinking ≥ 3 times /week %	13,298 (52.1)	4739 (48.0)	1041 (39.9)	15,254 (51.7)	2927 (47.3)	897 (39.0)	8578 (46.6)	9110 (54.0)	1390 (51.4)
Physical activity (MET-h/week) %	40.4 (40.4)	39.6 (39.9)	44.0 (46.2)	40.9 (41.1)	38.8 (39.2)	38.5 (39.4)	41.7 (42.6)	39.0 (38.4)	41.2 (41.2)
Body mass index (kg/m^2^)	28.2 (4.8)	28.5 (4.8)	29.3 (5.3)	27.9 (4.6)	29.3 (5.1)	31.0 (5.8)	28.6 (5.0)	28.1 (4.7)	28.1 (4.5)
Total energy (kJ/day)	8566 (2374)	9036 (2285)	9769 (2565)	8779 (2387)	8740 (2338)	8754 (2541)	8470 (2432)	8952 (2273)	9688 (2447)
Total sugar (g/day)	111.3 (44.7)	127.8 (43.9)	162.0 (54.8)	119.2 (47.1)	118.4 (45.9)	118.4 (52.6)	108.3 (46.2)	125.0 (42.9)	155.4 (54.8)
Fat (g/day)	74.8 (28.6)	79.2 (27.2)	82.6 (30.7)	76.4 (28.6)	76.6 (27.4)	77.8 (31.0)	75.3 (29.4)	77.3 (27.3)	79.5 (29.3)
Vegetable and fruits (servings/day)	5.0 (3.0)	4.8 (2.8)	4.6 (3.4)	4.9 (3.0)	4.8 (2.7)	4.8 (3.0)	4.8 (3.1)	4.9 (2.8)	5.3 (3.3)
Fish (servings/day)	2.0 (0.9)	1.9 (0.9)	1.8 (0.9)	2.0 (0.9)	1.9 (0.9)	1.9 (0.9)	1.9 (0.9)	2.0 (0.9)	2.0 (1.0)
Red meat (servings/day)	0.3 (0.2)	0.3 (0.2)	0.3 (0.2)	0.3 (0.2)	0.3 (0.2)	0.3 (0.2)	0.3 (0.2)	0.3 (0.2)	0.3 (0.2)
Insulin user %	330 (1.3)	104 (1.1)	24 (0.9)	287 (1.0)	104 (1.7)	67 (2.9)	276 (1.5)	170 (1.0)	12 (0.4)
Antihypertensive drug user %	14,060 (55.1)	5492 (55.7)	1430 (54.8)	16,356 (55.4)	3362 (54.3)	1264 (55.0)	9981 (54.2)	9467 (56.1)	1534 (56.7)
Aspirin user %	4798 (18.8)	1894 (19.2)	427 (16.4)	5549 (18.8)	1127 (18.2)	443 (19.3)	3339 (18.1)	3249 (19.3)	531 (19.6)
Lipid-lowering drug user %	7460 (29.2)	2918 (29.6)	728 (27.9)	8580 (29.1)	1848 (29.8)	678 (29.5)	5355 (29.1)	4948 (29.3)	803 (29.7)
Sugar-sweetened beverages (servings/day)	0.0 (0.0)	0.6 (0.3)	2.2 (0.9)	0.3 (0.6)	0.4 (0.6)	0.4 (0.8)	0.3 (0.7)	0.3 (0.6)	0.3 (0.7)
Artificially sweetened beverages (servings/day)	0.2 (0.6)	0.3 (0.5)	0.4 (0.8)	0.0 (0.0)	0.6 (0.3)	2.3 (1.0)	0.3 (0.7)	0.2 (0.6)	0.2 (0.6)
Pure fruit/vegetable juices (servings/day)	0.4 (0.6)	0.4 (0.5)	0.4 (0.7)	0.4 (0.6)	0.4 (0.5)	0.4 (0.6)	0.0 (0.0)	0.7 (0.3)	1.8 (0.7)

Values are expressed as mean (standard deviation) or number (percentage)

*MET* metabolic equivalents

*1 serving per day refers to 1 glass/can/carton/250mL per day

^a^Area-based Townsend deprivation index derived from consensus data on employment, housing, car ownership, and household overcrowding, corresponding to the postcode of residence

**Fig. 1:**
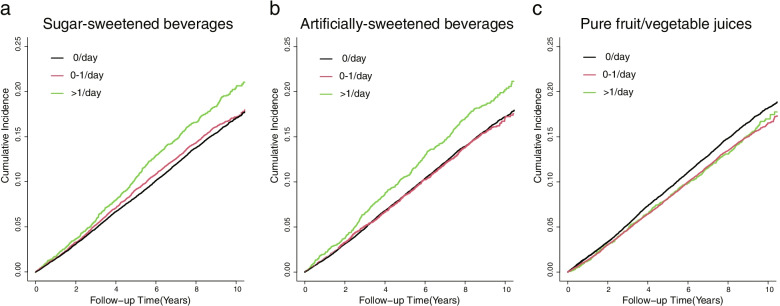
Cumulative incidence of CMM according to the consumption of sweet beverages. **a** The cumulative incidence of CMM in the highest SSBs consumption category (>1 serving per day) was higher than that in the lower consumption category in patients with single cardiometabolic disease. **b** The cumulative incidence of CMM in the highest ASBs consumption category (>1 serving per day) was also higher than that in the lower consumption category in patients with single cardiometabolic disease. **c** Participants who consumed pure fruit/vegetable juices (>1 serving per day and 0–1 serving per day) showed decreased cumulative incidence of CMM than those who did not. *CMM* cardiometabolic multimorbidity, *SSBs* sugar-sweetened beverages, *ASBs* artificially sweetened beverages

The cumulative incidence of CMM in the highest SSBs and ASBs consumption categories (>1 serving per day) was higher than that in the lower consumption category while participants who consumed pure fruit/vegetable juices showed a lower cumulative incidence of CMM than those who did not (Fig. 1). The cumulative incidence of CMM at the end of follow-up was 23.1 per 1000 person-year in the highest SSBs consumption category and 18.8 per 1000 person-year in participants who did not consume SSBs; 22.7 per 1000 person-year in the highest ASBs consumption category, 19.1 per 1000 person-year in participants who did not consume ASBs, 18.8 per 1000 person-year in the highest pure fruit/vegetable juices consumption category, and 20.3 per 1000 person-year in participants who did not consume pure fruit/vegetable juices. In the multivariable model, SSBs consumption was positively associated with the incidence of CMM (Table 2). Patients who consumed >1 serving per day had an 19% higher risk of CMM (HR: 1.19, 95% CI: 1.08–1.31) after adjusting for socioeconomic factors, BMI, lifestyle factors, dietary factors, and drug use. The highest consumption of ASBs (>1 serving per day) was also associated with a higher risk of CMM in model 3 (HR: 1.15, 95% CI: 1.04–1.27). Regarding pure fruit/vegetable juices, we found a lower risk of CMM in participants with moderate-level (0–1 serving per day) pure fruit/vegetable juice consumption (model 3: HR 0.90, 95% CI 0.85–0.94). High-level consumption of pure fruit/vegetable juices (>1 serving per day) showed marginal inverse association with CMM risks in model 3 (HR 0.90, 95% CI 0.81–0.99). However, the association of high-level consumption of pure fruit/vegetable juices became non-significant after Benjamini-Hochberg correction (adjusted *P*-value = 0.077, model 3).

**Table 2:** CMM risks according to the consumption of three types of beverages in UK Biobank at 2021 (*N* = 37,994)

	0/day HR (95% CI)	0–1/day HR (95% CI)	>1/day HR (95% CI)	Adjusted ***P***-trend^**a**^
**Sugar-sweetened beverages**				
Person-years	225,111	84,061	22,473	
Cases	4234	1648	517	
Model 0	1 (ref)	1.03 (0.97–1.09)	1.28 (1.17–1.41)	<0.001
Model 1	1 (ref)	1.01 (0.96–1.07)	1.19 (1.08–1.30)	0.003
Model 2	1 (ref)	1.01 (0.95–1.07)	1.18 (1.07–1.30)	0.005
Model 3	1 (ref)	1.01 (0.95-1.07)	1.19 (1.08-1.31)	0.005
**Artificially sweetened beverages**				
Person-years	258,806	53,106	19,733	
Cases	4952	999	448	
Model 0	1 (ref)	1.07 (1.01–1.15)	1.42 (1.29–1.57)	<0.001
Model 1	1 (ref)	0.99 (0.92–1.06)	1.19 (1.08–1.31)	0.007
Model 2	1 (ref)	0.98 (0.92–1.05)	1.19 (1.08–1.31)	0.008
Model 3	1 (ref)	0.97 (0.90–1.04)	1.15 (1.04–1.27)	0.045
**Pure fruit/vegetable juices**				
Person-years	162,028	146,007	23,610	
Cases	3283	2673	443	
Model 0	1 (ref)	0.85 (0.81–0.90)	0.87 (0.78–0.96)	<0.001
Model 1	1 (ref)	0.90 (0.85–0.94)	0.90 (0.82–0.99)	<0.001
Model 2	1 (ref)	0.89 (0.84–0.94)	0.89 (0.80–0.99)	<0.001
Model 3	1 (ref)	0.90 (0.85–0.94)	0.90 (0.81–0.99)	<0.001

Model 0: adjusted for age, sex, ethnicity, and deprivation index

Model 1: adjusted for variables in model 0 and smoking status, alcohol consumption, physical activity, sedentary time, and body mass index

Model 2: adjusted for variables in model 1 and total sugar intake, energy intake, fat intake, vegetable and fruit intake, fish intake, and red meat intake

Model 3: adjusted for variables in model 2 and insulin use, antihypertensive drug use, lipid-lowering drug use, and aspirin use

*CMM* cardiometabolic multimorbidity, *HR* hazard ratio, *CI* confidence interval, *ref* reference

^a^*P*-trend was adjusted with Benjamini-Hochberg correction

For participants with specific cardiometabolic disease, the highest consumption category of SSBs (>1 serving per day) had a 22% higher risk of CMM (HR: 1.22, 95% CI: 1.10–1.36) in patients with hypertension (Table 3). The association between SSBs consumption and CMM risk was not statistically significant in patients with cardiovascular disease and in patients with diabetes. We did not observe a positive association between ASBs intake and CMM risk among patients with specific cardiometabolic diseases. Pure fruit/vegetable juices demonstrated an inverse association with CMM in patients with hypertension who consumed 0–1 serving per day (HR: 0.91, 95% CI: 0.86–0.97). Consumption of pure fruit/vegetable juices (>0 serving per day) was inversely associated with CMM risks in patients with cardiovascular disease (HR: 0.79, 95% CI: 0.68–0.92). In patients with diabetes, pure fruit/vegetable consumption did not show inverse association with CMM risks.

**Table 3:** CMM risks in patients with the specific cardiometabolic disease according to the consumption of three types of beverages in UK Biobank at 2021 (*N* = 37,994)

		Numbers	Cases	HR (95% CI)
**Sugar-sweetened beverages**				
Hypertension	**0/day**	22,356	3262	1 (ref)
	**0**–**1/day**	8801	1336	1.04 (0.97–1.11)
	**>1/day**	2333	426	1.22 (1.10–1.36)
CVD	**0/day**	1625	499	1 (ref)
	**>0/day**	785	233	0.98 (0.83–1.15)
Diabetes	**0/day**	1534	473	1 (ref)
	**>0/day**	560	170	0.94 (0.78–1.13)
**Artificially sweetened beverages**				
Hypertension	**0/day**	26,134	3931	1 (ref)
	**0**–**1/day**	5413	774	0.96 (0.89–1.04)
	**>1/day**	1943	319	1.08 (0.96–1.22)
CVD	**0/day**	1970	599	1 (ref)
	**>0/day**	440	133	1.03 (0.85–1.25)
Diabetes	**0/day**	1397	422	1 (ref)
	**>0/day**	697	221	1.09 (0.92–1.29)
**Pure fruit/vegetable juices**				
Hypertension	**0/day**	15,991	2509	1 (ref)
	**0**–**1/day**	15,054	2146	0.91 (0.86–0.97)
	**>1/day**	2445	369	0.92 (0.82–1.03)
CVD	**0/day**	1181	396	1 (ref)
	**>0/day**	1229	336	0.79 (0.68–0.92)
Diabetes	**0/day**	1242	378	1 (ref)
	**>0/day**	852	265	1.00 (0.85–1.17)

Adjusted for age, sex, ethnicity, deprivation index, smoking status, alcohol consumption, physical activity, sedentary time, body mass index, total sugar intake, energy intake, fat intake, vegetable and fruit intake, fish intake, red meat intake, insulin use (diabetes only), antihypertensive drug use (hypertension only), lipid-lowering drug use, and aspirin use

*CMM* cardiometabolic multimorbidity, *HR* hazard ratio, *CI* confidence interval, *CVD* cardiovascular disease, *ref* reference

**Fig. 2:**
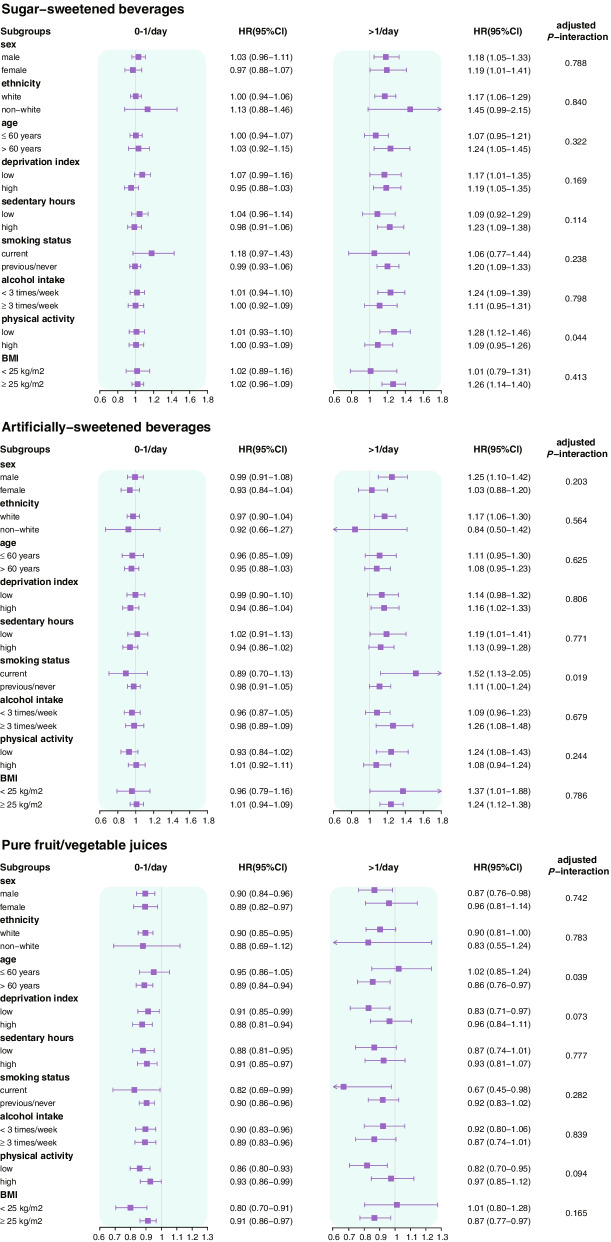
CMM risks stratified by potential risk factors according to the consumption of three types of beverages. Associations of three types of beverages’ consumption and risk of CMM were stratified by sex, ethnicity, age, deprivation index, sedentary hours, smoking status, alcohol intake, physical activity, BMI, and dietary pattern. The model was adjusted for age, sex, ethnicity and deprivation index, smoking status, alcohol consumption, physical activity, sedentary time and body mass index, total sugar intake, energy intake, fat intake, and healthy diet, insulin use, antihypertensive drug use, lipid-lowering drug use, and aspirin use. *CMM* cardiometabolic multimorbidity, *BMI* body mass index

Stratification analysis revealed an interaction between SSBs intake and physical activity (adjusted *P*-interaction = 0.044). For the association between ASBs and CMM, an interaction was observed for smoking status (adjusted *P*-interaction = 0.019). There was an interaction between the consumption of pure fruit/vegetable juices and age (adjusted *P*-interaction = 0.045). The additional stratified analysis using other covariates is presented in Fig. 2.

All eight sensitivity analyses were broadly consistent with the results of the main analysis, indicating the robustness of our findings (Additional files 4, 5, 6, 7, 8, 9, 10, and 11: Tables S3-S10). In the sixth sensitivity analysis, we recalculated the risks of CMM without adjusting for BMI to investigate the potential mediating effects of obesity (Additional file 9: Table S8). When BMI was excluded from the multivariate model, the HRs of CMM risks were higher for SSBs (>1 serving per day: HR: 1.25, 95% CI: 1.13–1.37) and ASBs consumptions (>1 serving per day: HR: 1.31, 95% CI: 1.18–1.44), but remained almost the same for pure fruit/vegetable consumption (>1 serving per day: HR: 0.89, 95% CI: 0.81–0.99). In the sensitivity analysis using the MSM model, we also found no major differences from our main findings, further proving the stability of our results (Additional file 11: Table S10).

## Discussion

In this prospective cohort of the UK Biobank, consumption of SSBs and ASBs was associated with a higher risk of CMM in patients with a single cardiometabolic disease, while pure fruit/vegetable juice intake was inversely associated with the risk of CMM. These associations were independent of obesity and other dietary factors including energy, fat, and sugar intake. We also observed a positive association between SSBs consumption and CMM risks in participants with hypertension and an inverse association between moderate-level pure fruit/vegetable juice consumption and CMM risk in participants with hypertension and in participants with cardiovascular disease (stroke or coronary heart disease).

To the best of our knowledge, this is the first cohort study to investigate the impact of SSBs consumption on CMM risk in patients with a single cardiometabolic disease. Previous studies focused mainly on the general healthy population; therefore, direct comparison of our results with existing evidence could not be done. Nonetheless, our findings are consistent with those of previous studies in different populations. Recent meta-analyses suggested that a serving per day increment in SSBs consumption was associated with an 8–20% higher risk of hypertension, diabetes, or cardiovascular disease [21, 24]. In a cohort of 106,178 women, participants who consumed over 1 serving of SSBs per day had a 21% higher risk of stroke compared with non-consumers [25]. Our study similarly showed that SSBs consumption of more than one serving per day was associated with an 19% higher risk of CMM. This indicated that the role of SSBs intake in the secondary prevention of cardiometabolic diseases was as notable as its role in primary prevention. Although it is generally assumed that patients with a single cardiometabolic disease tend to make healthier lifestyle choices than the general population, we found that the consumption patterns of these three types of beverages were comparable. SSBs were consumed by 32.8% of participants included in our analysis, 22.4% for ASBs, and 51.5% for pure fruit/vegetable juices, while 32.8% for SSBs, 20.6% for ASBs, and 52.2% for pure fruit/vegetable juices occurred in the general UK Biobank population [33].

In the analysis for patients with specific cardiometabolic diseases, SSBs consumption was associated with a higher risk of CMM in hypertensive patients but not in patients with diabetes and in patients with cardiovascular diseases. A cross-sectional study found a positive association between SSBs intake and hypertension in patients with type 2 diabetes [14]; however, this study was limited by small sample size (*N* = 157) and was not adjusted for medication use. The confounding effects on drug use in the relationship between SSBs consumption and CMM risk might be partly explained by the biological mechanism through which SSBs contribute to cardiometabolic disease. SSBs intake induces rapid spikes in postprandial blood glucose and insulin levels [41]. In addition, sucrose and fructose in SSBs can increase blood triglyceride levels and hepatic de novo lipogenesis [42]. Diabetic patients are encouraged to monitor and control their blood glucose levels at a normal level using insulin and/or oral medications. Similarly, patients with cardiovascular disease are recommended to reduce their blood lipids to lower levels than those in the general population. Medication use in patients with diabetes and cardiovascular disease might alleviate metabolic disorders of blood sugar and lipids induced by SSBs. Nevertheless, SSBs consumption was associated with a higher risk of CMM in hypertensive patients and might still be notable in patients without sustained blood glucose and lipid control. Our findings support stricter restrictions for excessive SSBs consumption in the present lifestyle intervention guidelines for cardiometabolic patients, especially those with hypertension.

ASBs are assumed to be a healthier alternative for people who should avoid excessive calories in SSBs. There is also no available evidence on the relationship between ASBs consumption and CMM risks. We observed that consumption of ASBs was associated with a higher risk of CMM, which is consistent with the positive associations observed in several studies between ASBs intake and the incidence of type 2 diabetes [21, 22], hypertension [21], and cardiovascular disease [24, 29] in healthy people. Several reports of artificial sweeteners that could alter the gut microbiota and induce insulin resistance might be related to the underlying explanation for the impact of ASBs consumption on CMM risks [43, 44]. However, ASBs intake was not associated with CMM risk in the analysis for patients with specific cardiometabolic diseases. Thus, ASBs consumption may be representative of a poor lifestyle. Although we adjusted for numerous potential confounders, the association between ASBs and CMM could still be confounded by other unknown comorbidities in patients with a single cardiometabolic disease. Furthermore, patients with a higher risk of cardiometabolic diseases, such as obese individuals, are more likely to switch from SSBs to ASBs consumption. However, these findings should be cautiously interpreted. The sample size in the analysis for diabetic patients (*N* = 2094) and for patients with cardiovascular disease (*N* = 2410) was relatively small. This might be one of the possible explanations for the non-significant associations and the wide 95% CI range. Nevertheless, ASBs should not be recommended as a safe alternative to provide sweet flavour for patients with cardiometabolic diseases.

Few studies have examined the effect of pure fruit juices on cardiometabolic outcomes. A large-sample cohort study that was conducted in the UK Biobank population reported inverse associations between pure fruit/vegetable juice consumption and all-cause mortality [33]. Similarly, our study showed inverse associations between pure fruit/vegetable juices and the incidence of CMM. A recent meta-analysis showed a biphasic dose-response relationship in cardiovascular disease and hypertension, where inverse associations were observed at low-moderate levels of 100% fruit juice consumption but disappeared at high-level consumption [19, 34]. We also found that only moderate-level consumption of pure fruit/vegetable juices (0–1 serving/day) was inversely associated with CMM risk in patients with hypertension. This may be explained by the beneficial effects of vitamins and other bioactive substances in fruit juices. However, these benefits may be offset by the harmful consequences of rapidly absorbed sugar and liquid energy at higher levels of consumption. Previous studies have not demonstrated the beneficial effects of fruit juices on diabetes [30, 34]. Also, the analysis for diabetic patients did not show an inverse association of fruit juices, probably because diabetic patients are more sensitive to the unfavourable effects of excess sugar.

The strengths of our study include its large sample size, prospective design, and long follow-up time. The detailed socioeconomic, behavioural, and clinical profile data in the UK Biobank allowed us to access specific exposure information and to adjust numerous potential confounders. Validated end-point events were ascertained by linkage to primary care, death register, and hospitalisation records, enhancing the reliability and reducing bias caused by loss to follow-up. In addition, our study investigated the association between the consumption of three types of beverages and progression to CMM in individuals with a single cardiometabolic disease. Previous studies on multimorbidity risk have often investigated the progression to multimorbidity in participants with and without a single non-communicable disease, which may confuse the results because the impact of risk factors might be different in these two groups [12].

The findings of this study should be interpreted with caution considering these limitations. First, as an observational study, we could only determine the association, not causation since the possibility of residual confounding and reverse causation is always possible. Second, dietary data and other covariates were not simultaneously collected. We conducted a sensitivity analysis using baseline questionnaire dietary data only, to minimise this effect. The results show no major differences with the main findings. Third, the fact that only 60.1% of participants completed the questionnaire on two or more occasions might limit the reliability because dietary intake from two dietary assessments is closer to the usual intake than a single assessment [45, 46]. However, our sensitivity analysis using baseline questionnaire dietary data only also produced persistent findings. Fourth, the beverage intake assessment was carried out in the first 2 years at baseline; therefore, we could not rule out the possibility of modifications of dietary habits during follow-up. Future research using regular dietary assessments may minimise the impact of dietary changes. Fifth, the sample size in the analysis for patients with the specific cardiometabolic disease was relatively small, especially for diabetic patients (*N* = 2094) and for patients with cardiovascular disease (*N*=2410). Finally, our study was performed among predominantly Caucasian Europeans, which may limit its generalisability.

## Conclusions

Our study showed that a higher consumption of SSBs was associated with a higher risk of CMM in individuals with a single cardiometabolic disease, while pure fruit/vegetable juice intake showed an inverse association with the incidence of CMM. We also found that ASBs intake may be a risk factor for CMM, which should be carefully interpreted considering residual confounding and reverse causation. Our findings highlight the need for further restriction of SSBs and ASBs intake to improve secondary prevention of cardiometabolic diseases.

## Supplementary Information

**Additional file 1: Figure S1**. Study population flow chart. For 502,414 UK Biobank participants, we included participants who completed the online 24-h dietary recall questionnaire on at least one occasion and also had a history of a single cardiometabolic disease (coronary heart disease, hypertension, stroke, or diabetes) at baseline. We excluded participants who had missing data or who reported dubious intake of total energy, defined as < 500 or >3500 kcal/d for women and <800 or >4200 kcal/day for men as previous study, leaving 37,994 participants for analysis.

**Additional file 2: Table S1**. Detailed information about covariate variables in UK Biobank at 2021 (*N*=37,994). The detailed information about all covariate variables in the analysis, including the definition, types and the number of categories.

**Additional file 3: Table S2**. Specific diagnostic criteria for coronary heart disease, hypertension, stroke, and diabetes in UK Biobank at 2021 (*N*=37,994). The specific diagnostic criteria for coronary heart disease, hypertension, stroke, and diabetes in our study included self-reported diagnosis, medication history, surgery history, and electronic health record coded by International Classification of Diseases version 9 (ICD-9), International Classification of Diseases version 10 (ICD-10), and Office of Population Censuses and Surveys Classification of Interventions and Procedures version 4 (OPCS-4).

**Additional file 4: Table S3**. CMM risks eliminating first two years of follow-up in UK Biobank at 2021 (*N*=36,555). We eliminate the first 2 years of follow-up to minimize the reverse causality. *CMM cardiometabolic multimorbidity (DOCX 21 kb)*

**Additional file 5: Table S4**. CMM risks excluding participants who lost weight compared to one year before recruitment in UK Biobank at 2021 (*N*=31,193). We recalculated the risks after excluding participants who reported losing weight compared with 1 year before recruitment in a baseline touchscreen questionnaire to decrease the effects of going on a diet. *CMM cardiometabolic multimorbidity (DOCX 21 kb)*

**Additional file 6: Table S5**. CMM risks using the beverage consumption from the baseline questionnaire in UK Biobank at 2021 (*N*=13,284). We re-ran the model using the beverages intake ascertained only from the baseline questionnaire instead of the mean intake of all questionnaires completed to approach baseline status. *CMM cardiometabolic multimorbidity (DOCX 22 kb)*

**Additional file 7: Table S6**. CMM risks with adjustments for alcohol consumption frequency or alcohol consumption units in UK Biobank at 2021 (*N*=37,994). To test for residual confounding of alcohol consumption, we re-ran the models replacing alcohol consumption frequency (drinking ≥ 3 times per week or not) with alcohol consumption units (≥14 units per week or not). *CMM cardiometabolic multimorbidity (DOCX 19 kb)*

**Additional file 8: Table S7**. CMM risks using a different approach to assess the level of physical activity in UK Biobank at 2021 (*N*=37,994). We recalculated the models using different approach to assess the physical activity. We divided participants into two groups based on whether they met the 2017 UK Physical activity guidelines of 150 minutes of walking or moderate activity per week or 75 minutes of vigorous activity. *CMM cardiometabolic multimorbidity (DOCX 19 kb)*

**Additional file 9: Table S8**. CMM risks with or without adjustments for body mass index in UK Biobank at 2021 (*N*=37,994). To investigate whether the effects of three types of beverages’ consumption were partially mediated via obesity, we re-ran the models without adjusting for BMI. *CMM cardiometabolic multimorbidity, BMI body mass index (DOCX 19 kb)*

**Additional file 10: Table S9**. CMM risks with or without mutual adjustments for other kinds of beverages’ consumption in UK Biobank at 2021 (*N*=37,994). To minimize the confounding effects of other beverages consumption, we re-run the analysis mutually adjusted for 3 different types of beverages, i.e., SSBs were adjusted for ASBs and pure fruit/vegetable juices intake, and vice versa. *CMM cardiometabolic multimorbidity, SSBs sugar-sweetened beverages, ASBs artificially-sweetened beverages (DOCX 19 kb)*

**Additional file 11: Table S10**. CMM risks according to the consumption of three types of beverages using multi-state model in UK Biobank at 2021 (*N*=119,589). Using multi-state model to recalculate the associations between three types of beverages and cardiometabolic outcomes, which was the classical and reliable approach for multimorbidity study. The MSM approach allowed simultaneous estimation of the role of risk factors in the transitions, which included from healthy to single cardiometabolic disease and from single cardiometabolic disease to cardiometabolic multimorbidity. *CMM cardiometabolic multimorbidity*.

## Data Availability

The data that support the findings of this study are available from UK Biobank project site. All bona fide researchers can apply to access and use the UK Biobank resource at http://www.ukbiobank.ac.uk. This research has been conducted using the UK Biobank Resource under Application Number 76118.

## Abbreviations

ASBs: Artificially sweetened beverages
BMI: Body mass index
CI: Confidence interval
CMM: Cardiometabolic multimorbidity
HR: Hazard ratio
IPAQ: International Physical Activity Questionnaire
IQR: Interquartile range
NHS: National Health Service
SSBs: Sugar-sweetened beverages
