# Supplementary figures and images for: Associations between consumption of three types of beverages and risk of cardiometabolic multimorbidity in UK Biobank participants: a prospective cohort study
Source: BMC Med. 2022 Aug 18;20:273. doi: 10.1186/s12916-022-02456-4 (PMC9386995; doi:10.1186/s12916-022-02456-4)

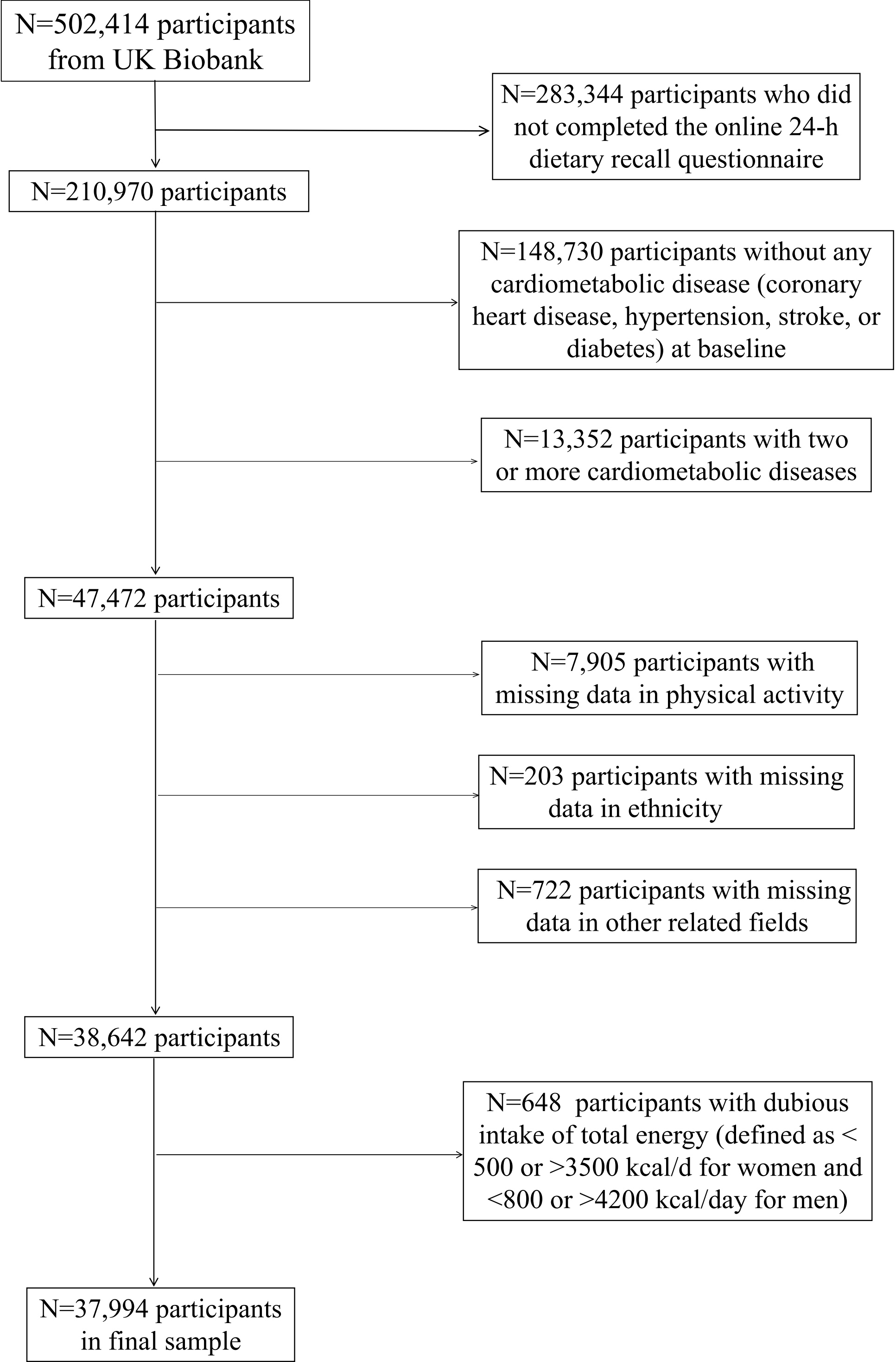

Supplement: Supplementary file 1 — Additional file 1: Figure S1. Study population flow chart. For 502,414 UK Biobank participants, we included participants who completed the online 24-h dietary recall questionnaire on at least one occasion and also had a history of a single cardiometabolic disease (coronary heart disease, hypertension, stroke, or diabetes) at baseline. We excluded participants who had missing data or who reported dubious intake of total energy, defined as < 500 or >3500 kcal/d for women and <800 or >4200 kcal/day for men as previous study, leaving 37,994 participants for analysis. [file 12916_2022_2456_MOESM1_ESM.pdf]
