# Supplementary material for: Associations between consumption of three types of beverages and risk of cardiometabolic multimorbidity in UK Biobank participants: a prospective cohort study
Source: BMC Med. 2022 Aug 18;20:273. doi: 10.1186/s12916-022-02456-4 (PMC9386995; doi:10.1186/s12916-022-02456-4)
Supplement: Supplementary file 2 — Additional file 2: Table S1. Detailed information about covariate variables in UK Biobank at 2021 (N=37,994). The detailed information about all covariate variables in the analysis, including the definition, types and the number of categories. [file 12916_2022_2456_MOESM2_ESM.docx]

**Table** **S1 Detailed information about covariate variables in UK Biobank at 2021 (N=37,994)**

| **Covariate** | **Description** | **Types** | **Category** |
| --- | --- | --- | --- |
| Gender | Gender of the participants | Categorical variable | Group 1: Male |
|  |  |  | Group 2: Female |
| Ethnicity | Ethnicity of the participants | Categorical variable | Group 1: White |
|  |  |  | Group 2: Non-white |
| Age | Age of the participants | Continuous variable | / |
| Deprivation index | Area-based Townsend deprivation index derived from consensus data on employment, housing, car ownership, and household overcrowding, corresponding to the postcode of residence | Continuous variable | / |
| Sedentary hours | Summed time using computer, driving, and watching television per day | Continuous variable | / |
| Smoking status | Current/past smoking status of participants | Categorical variable | Group 1: Current smokers |
|  |  |  | Group 2: Previous or non-smokers |
| Alcohol  Consumption | Weekly alcohol consumption frequency | Categorical variable | Group 1: Drinking ≥ 3 times per week |
|  |  |  | Group 2: Drinking < 3 times per week |
| Alcohol  Consumption units (in Supplemental Table 6) | Weekly average alcohol consumption units obtained by multiplying the average weekly red wine, champagne, beer, spirits and fortified wine intake by corresponding alcohol units | Categorical variable | Group 1: Drinking ≥ 14 units per week |
|  |  |  | Group 2: Drinking < 14 units per week |
| Physical activity | Summed metabolic equivalents (MET-h/week) which were calculated by multiplying the duration of light, moderate and vigorous physical activity and weights of 2.5, 4, and 8, respectively, and then summing them. | Continuous variable | / |
| Physical activity  (in Supplemental Table 5) | Whether the participants meeting the 2017 UK Physical activity guidelines of 150 minutes of walking or moderate activity per week or 75 minutes of vigorous activity | Categorical variable | Group 1: Meeting the standard |
|  |  |  | Group 2: Not meeting the standard |
| BMI | BMI of the participants | Continuous variable | / |
| Total energy | Calculated total energy from the dietary questionnaire | Continuous variable | / |
| Total sugar | Calculated total sugar from the dietary questionnaire | Continuous variable | / |
| Fat | Calculated fat from the dietary questionnaire | Continuous variable | / |
| Vegetable and fruit intake | Summed vegetable and fruit intake per day from the dietary questionnaire | Continuous variable | / |
| Fish intake | Summed fish intake per day from the dietary questionnaire | Continuous variable | / |
| Red meat intake | Summed red meat intake per day from the dietary questionnaire | Continuous variable | / |
| Insulin user | Whether the participants had a history of using insulin | Categorical variable | Group 1: Having the history of using insulin |
|  |  |  | Group 2: Not having the history of using insulin |
| Antihypertensive drugs user | Whether the participants had a history of using antihypertensive drugs | Categorical variable | Group 1: Having the history of using antihypertensive drugs |
|  |  |  | Group 2: Not having the history of using antihypertensive drugs |
| Aspirin user | Whether the participants had a history of using aspirin | Categorical variable | Group 1: Having the history of using aspirin |
|  |  |  | Group 2: Not having the history of using aspirin |
| Lipid-lowering drugs user | Whether the participants had a history of using lipid-lowering drugs | Categorical variable | Group 1: Having the history of using lipid-lowering drugs |
|  |  |  | Group 2: Not having the history of using lipid-lowering drugs |

BMI body mass index.
