# Supplementary material for: Associations between consumption of three types of beverages and risk of cardiometabolic multimorbidity in UK Biobank participants: a prospective cohort study
Source: BMC Med. 2022 Aug 18;20:273. doi: 10.1186/s12916-022-02456-4 (PMC9386995; doi:10.1186/s12916-022-02456-4)
Supplement: Supplementary file 3 — Additional file 3: Table S2. Specific diagnostic criteria for coronary heart disease, hypertension, stroke, and diabetes in UK Biobank at 2021 (N=37,994). The specific diagnostic criteria for coronary heart disease, hypertension, stroke, and diabetes in our study included self-reported diagnosis, medication history, surgery history, and electronic health record coded by International Classification of Diseases version 9 (ICD-9), International Classification of Diseases version 10 (ICD-10), and Office of Population Censuses and Surveys Classification of Interventions and Procedures version 4 (OPCS-4). [file 12916_2022_2456_MOESM3_ESM.docx]

**Table S2 Specific diagnostic criteria for coronary heart disease, hypertension, stroke and diabetes in UK Biobank at 2021 (N=37,994)**

| **Disease** | Coronary heart disease | Hypertension | Diabetes | Stroke |
| --- | --- | --- | --- | --- |
| **Self-report** | angina and myocardial infarction/heart attack self-report | hypertension self-report | diabetes self-report | stroke self-report |
| **Medication history** | / | blood pressure medication | insulin | / |
| **Surgery history** | coronary angioplasty, coronary artery bypass grafts and triple heart bypass | / | / | / |
| **ICD-9** | 410-414 | 401-405 | 250, 3572, 3620 | 3361, 36231, 36232, 430, 431, 4329, 43301, 43311, 43321, 43331, 43381, 43391, 434, 436 |
| **ICD-10** | I20-I25, Z95.1, Z95.5 | I10-I13, I15, O10 | E10-E14, G59.0, G63.2, H28.0, H36.0, M14.2, N08.3 | I60, I61, I62.9, I63, I64, I67.8, I69.0, I69.3, G95.1, H34.1, H34.2, S06.6 |
| **OPCS-4** | K40-K46, K49, K50, K75 | / | / | A05.2-A05.4, L35.1, L35.3, L34.3 |

ICD-9 International Classification of Diseases version 9; ICD-10 International Classification of Diseases version 10; OPCS-4 Office of Population Censuses and Surveys Classification of Interventions and Procedures version 4
