# Supplementary material for: Associations between consumption of three types of beverages and risk of cardiometabolic multimorbidity in UK Biobank participants: a prospective cohort study
Source: BMC Med. 2022 Aug 18;20:273. doi: 10.1186/s12916-022-02456-4 (PMC9386995; doi:10.1186/s12916-022-02456-4)
Supplement: Supplementary file 4 — Additional file 4: Table S3. CMM risks eliminating first two years of follow-up in UK Biobank at 2021 (N=36,555). We eliminate the first 2 years of follow-up to minimize the reverse causality. CMM cardiometabolic multimorbidity (DOCX 21 kb) [file 12916_2022_2456_MOESM4_ESM.docx]

**Table S3 CMM risks eliminating first two years of follow-up in UK Biobank at 2021 (N=36,555)**

|  | | **0/day**  **HR (95% CI)** | **0-1/day**  **HR (95% CI)** | **>1/day**  **HR (95% CI)** | ***P* value**  **for trend** |
| --- | --- | --- | --- | --- | --- |
| **Sugar-sweetened beverages** | | | | | |
|  | Person-years | 224167 | 83651 | 22354 |  |
|  | Cases | 3449 | 1323 | 420 |  |
|  | Model 0 | 1(ref) | 1.03 (0.96-1.09) | 1.29 (1.17-1.43) | <0.001 |
|  | Model 1 | 1(ref) | 1.01 (0.94-1.07) | 1.19 (1.08-1.32) | 0.002 |
|  | Model 2 | 1(ref) | 1.00 (0.94-1.07) | 1.18 (1.06-1.32) | 0.004 |
|  | Model 3 | 1(ref) | 1.00 (0.94-1.07) | 1.19 (1.07-1.32) | 0.004 |
| **Artificially-sweetened beverages** | | | | | |
|  | Person-years | 257667 | 52861 | 19644 |  |
|  | Cases | 4034 | 796 | 362 |  |
|  | Model 0 | 1(ref) | 1.05 (0.98-1.14) | 1.41 (1.27-1.58) | <0.001 |
|  | Model 1 | 1(ref) | 0.97 (0.90-1.05) | 1.19 (1.062-1.33) | 0.009 |
|  | Model 2 | 1(ref) | 0.97 (0.90-1.05) | 1.19 (1.06-1.32) | 0.010 |
|  | Model 3 | 1(ref) | 0.95 (0.88-1.03) | 1.15 (1.03-1.28) | 0.041 |
| **Pure fruit/vegetable juices** | | | | | |
|  | Person-years | 161312 | 145356 | 23504 |  |
|  | Cases | 2667 | 2165 | 360 |  |
|  | Model 0 | 1(ref) | 0.86 (0.81-0.91) | 0.87 (0.78-0.97) | <0.001 |
|  | Model 1 | 1(ref) | 0.90 (0.85-0.95) | 0.90 (0.80-0.99) | 0.003 |
|  | Model 2 | 1(ref) | 0.89 (0.84-0.95) | 0.88(0.79-0.99) | 0.001 |
|  | Model 3 | 1(ref) | 0.90 (0.85-0.95) | 0.89 (0.79-0.99) | 0.002 |

CMM cardiometabolic multimorbidity; HR hazard ratio; CI confidence interval; ref reference

Model 0: adjusted for age, sex, ethnicity, and deprivation index

Model 1: adjusted for variables in model 0 and smoking status, alcohol consumption, physical activity, sedentary time, and body mass index

Model 2: adjusted for variables in model 1 and total sugar intake, energy intake, fat intake, vegetable and fruit intake, fish intake, and red meat intake

Model 3: adjusted for variables in model 2 and insulin use, antihypertensive drugs use, lipid-lowering drugs use, and aspirin use
