# Supplementary material for: Associations between consumption of three types of beverages and risk of cardiometabolic multimorbidity in UK Biobank participants: a prospective cohort study
Source: BMC Med. 2022 Aug 18;20:273. doi: 10.1186/s12916-022-02456-4 (PMC9386995; doi:10.1186/s12916-022-02456-4)
Supplement: Supplementary file 5 — Additional file 5: Table S4. CMM risks excluding participants who lost weight compared to one year before recruitment in UK Biobank at 2021 (N=31,193). We recalculated the risks after excluding participants who reported losing weight compared with 1 year before recruitment in a baseline touchscreen questionnaire to decrease the effects of going on a diet. CMM cardiometabolic multimorbidity (DOCX 21 kb) [file 12916_2022_2456_MOESM5_ESM.docx]

**Table S4 CMM risks excluding participants who lost weight compared to one year before recruitment in UK Biobank at 2021 (N=31,193)**

|  | | **0/day**  **HR (95%CI)** | **0-1/day**  **HR (95%CI)** | **>1/day**  **HR (95%CI)** | ***P* value**  **for trend** |
| --- | --- | --- | --- | --- | --- |
| **Sugar-sweetened beverages** | | | | | |
|  | Person-years | 184416 | 69737 | 18276 |  |
|  | Cases | 3425 | 1378 | 411 |  |
|  | Model 0 | 1(ref) | 1.06 (0.99-1.13) | 1.28 (1.15-1.41) | <0.001 |
|  | Model 1 | 1(ref) | 1.04 (0.97-1.10) | 1.18 (1.06-1.31) | 0.002 |
|  | Model 2 | 1(ref) | 1.04 (0.97-1.10) | 1.19 (1.06-1.32) | 0.002 |
|  | Model 3 | 1(ref) | 1.04 (0.97-1.10) | 1.19 (1.07-1.32) | 0.002 |
| **Artificially-sweetened beverages** | | | | | |
|  | Person-years | 215803 | 42101 | 14525 |  |
|  | Cases | 4080 | 806 | 328 |  |
|  | Model 0 | 1(ref) | 1.10 (1.02-1.19) | 1.42 (1.27-1.59) | <0.001 |
|  | Model 1 | 1(ref) | 1.01 (0.93-1.09) | 1.17 (1.05-1.32) | 0.001 |
|  | Model 2 | 1(ref) | 1.01 (0.93-1.09) | 1.17 (1.05-1.32) | 0.001 |
|  | Model 3 | 1(ref) | 0.99 (0.92-1.07) | 1.14 (1.01-1.28) | 0.005 |
| **Pure fruit/vegetable juices** | | | | | |
|  | Person-years | 131123 | 121895 | 19411 |  |
|  | Cases | 2678 | 2195 | 361 |  |
|  | Model 0 | 1(ref) | 0.85 (0.80-0.90) | 0.86 (0.76-0.95) | <0.001 |
|  | Model 1 | 1(ref) | 0.89 (0.84-0.94) | 0.89 (0.79-0.99) | 0.004 |
|  | Model 2 | 1(ref) | 0.88 (0.84-0.94) | 0.89 (0.78-0.99) | 0.004 |
|  | Model 3 | 1(ref) | 0.89 (0.84-0.94) | 0.89(0.79-0.99) | 0.006 |

CMM cardiometabolic multimorbidity; HR hazard ratio; CI confidence interval; ref reference

Model 0: adjusted for age, sex, ethnicity, and deprivation index

Model 1: adjusted for variables in model 0 and smoking status, alcohol consumption, physical activity, sedentary time, and body mass index

Model 2: adjusted for variables in model 1 and total sugar intake, energy intake, fat intake, vegetable and fruit intake, fish intake, and red meat intake

Model 3: adjusted for variables in model 2 and insulin use, antihypertensive drugs use, lipid-lowering drugs use, and aspirin use
