# Supplementary material for: Associations between consumption of three types of beverages and risk of cardiometabolic multimorbidity in UK Biobank participants: a prospective cohort study
Source: BMC Med. 2022 Aug 18;20:273. doi: 10.1186/s12916-022-02456-4 (PMC9386995; doi:10.1186/s12916-022-02456-4)
Supplement: Supplementary file 6 — Additional file 6: Table S5. CMM risks using the beverage consumption from the baseline questionnaire in UK Biobank at 2021 (N=13,284). We re-ran the model using the beverages intake ascertained only from the baseline questionnaire instead of the mean intake of all questionnaires completed to approach baseline status. CMM cardiometabolic multimorbidity (DOCX 22 kb) [file 12916_2022_2456_MOESM6_ESM.docx]

**Table S5 CMM risks using the beverage consumption from the baseline questionnaire in UK Biobank at 2021 (N=13,284)**

|  | | **0/day**  **HR (95% CI)** | **0-1/day**  **HR (95% CI)** | **>1/day**  **HR (95% CI)** | ***P* value**  **for trend** |
| --- | --- | --- | --- | --- | --- |
| **Sugar-sweetened beverages** | | | | | |
|  | Person-years | 10507 | 18947 | 9259 |  |
|  | Cases | 1956 | 336 | 207 |  |
|  | Model 0 | 1 (ref) | 0.97 (0.86-1.09) | 1.28 (1.10-1.47) | 0.018 |
|  | Model 1 | 1 (ref) | 0.93 (0.82-1.04) | 1.19 (1.03-1.37) | 0.023 |
|  | Model 2 | 1 (ref) | 0.91 (0.81-1.03) | 1.16 (1.01-1.35) | 0.040 |
|  | Model 3 | 1 (ref) | 0.93 (0.82-1.04) | 1.16 (1.01-1.35) | 0.034 |
| **Artificially-sweetened beverages** | | | | | |
|  | Person-years | 11394 | 12484 | 6722 |  |
|  | Cases | 2129 | 212 | 158 |  |
|  | Model 0 | 1 (ref) | 1.05 (0.91-1.20) | 1.52 (1.29-1.79) | <0.001 |
|  | Model 1 | 1 (ref) | 0.96 (0.83-1.11) | 1.30 (1.10-1.53) | 0.023 |
|  | Model 2 | 1 (ref) | 0.96 (0.83-1.11) | 1.30 (1.10-1.53) | 0.024 |
|  | Model 3 | 1 (ref) | 0.96 (0.83-1.10) | 1.26 (1.07-1.49) | 0.005 |
| **Pure fruit/vegetable juices** | | | | | |
|  | Person-years | 81449 | 45259 | 8592 |  |
|  | Cases | 1579 | 775 | 145 |  |
|  | Model 0 | 1 (ref) | 0.86 (0.79-0.93) | 0.85 (0.76-0.97) | 0.008 |
|  | Model 1 | 1 (ref) | 0.89 (0.81-0.97) | 0.86 (0.76-0.97) | 0.019 |
|  | Model 2 | 1 (ref) | 0.88 (0.81-0.96) | 0.88 (0.77-0.96) | 0.015 |
|  | Model 3 | 1 (ref) | 0.88 (0.86-0.94) | 0.89 (0.76-0.99) | 0.013 |

CMM cardiometabolic multimorbidity; HR hazard ratio; CI confidence interval; ref reference

Model 0: adjusted for age, sex, ethnicity, and deprivation index

Model 1: adjusted for variables in model 0 and smoking status, alcohol consumption, physical activity, sedentary time, and body mass index

Model 2: adjusted for variables in model 1 and total sugar intake, energy intake, fat intake, vegetable and fruit intake, fish intake, and red meat intake

Model 3: adjusted for variables in model 2 and insulin use, antihypertensive drugs use, lipid-lowering drugs use, and aspirin use
