# Supplementary material for: Associations between consumption of three types of beverages and risk of cardiometabolic multimorbidity in UK Biobank participants: a prospective cohort study
Source: BMC Med. 2022 Aug 18;20:273. doi: 10.1186/s12916-022-02456-4 (PMC9386995; doi:10.1186/s12916-022-02456-4)
Supplement: Supplementary file 7 — Additional file 7: Table S6. CMM risks with adjustments for alcohol consumption frequency or alcohol consumption units in UK Biobank at 2021 (N=37,994). To test for residual confounding of alcohol consumption, we re-ran the models replacing alcohol consumption frequency (drinking ≥ 3 times per week or not) with alcohol consumption units (≥14 units per week or not). CMM cardiometabolic multimorbidity (DOCX 19 kb) [file 12916_2022_2456_MOESM7_ESM.docx]

**Table S6 CMM risks with adjustments for alcohol consumption frequency or alcohol consumption units in UK Biobank at 2021 (N=37,994)**

|  | | **0/day**  **HR (95% CI)** | **0-1/day**  **HR (95% CI)** | **>1/day**  **HR (95% CI)** | ***P* value**  **for trend** |
| --- | --- | --- | --- | --- | --- |
| **Sugar-sweetened beverages** | | | | | |
|  | Model 3 | 1 (ref) | 1.01 (0.95-1.07) | 1.19 (1.08-1.31) | 0.005 |
|  | Model 3 (A) | 1 (ref) | 0.99 (0.93-1.07) | 1.14 (1.01-1.28) | 0.009 |
| **Artificially-sweetened beverages** | | | | | |
|  | Model 3 | 1 (ref) | 0.97 (0.90-1.04) | 1.15 (1.04-1.27) | 0.045 |
|  | Model 3 (A) | 1 (ref) | 0.97 (0.89-1.05) | 1.13 (1.01-1.27) | 0.015 |
| **Pure fruit/vegetable juices** | | | | | |
|  | Model 3 | 1 (ref) | 0.90 (0.85-0.94) | 0.90 (0.81-0.99) | <0.001 |
|  | Model 3 (A) | 1 (ref) | 0.88 (0.83-0.94) | 0.84 (0.74-0.96) | <0.001 |

CMM cardiometabolic multimorbidity; HR hazard ratio; CI confidence interval; ref reference

Model 3: adjusted for age, sex, ethnicity, deprivation index, smoking status, alcohol consumption units, physical activity, sedentary time, body mass index, total sugar intake, energy intake, fat intake, vegetable and fruit intake, fish intake, red meat intake, insulin use, antihypertensive drugs use, lipid-lowering drugs use, and aspirin use

Model 3(A): adjusted for variables in model 3 and replaced alcohol consumption frequency with alcohol consumption units
