# Supplementary material for: Associations between consumption of three types of beverages and risk of cardiometabolic multimorbidity in UK Biobank participants: a prospective cohort study
Source: BMC Med. 2022 Aug 18;20:273. doi: 10.1186/s12916-022-02456-4 (PMC9386995; doi:10.1186/s12916-022-02456-4)
Supplement: Supplementary file 8 — Additional file 8: Table S7. CMM risks using a different approach to assess the level of physical activity in UK Biobank at 2021 (N=37,994). We recalculated the models using different approach to assess the physical activity. We divided participants into two groups based on whether they met the 2017 UK Physical activity guidelines of 150 minutes of walking or moderate activity per week or 75 minutes of vigorous activity. CMM cardiometabolic multimorbidity (DOCX 19 kb) [file 12916_2022_2456_MOESM8_ESM.docx]

**Table S7 CMM risks using a different approach to assess the level of physical activity in UK Biobank at 2021 (N=37,994)**

|  | | **0/day**  **HR (95% CI)** | **0-1/day**  **HR (95% CI)** | **>1/day**  **HR (95% CI)** | ***P* value**  **for trend** |
| --- | --- | --- | --- | --- | --- |
| **Sugar-sweetened beverages** | | | | | |
|  | Model 3 | 1 (ref) | 1.01 (0.95-1.07) | 1.19 (1.08-1.31) | 0.005 |
|  | Model 3 (P) | 1 (ref) | 1.01 (0.95-1.06) | 1.19 (1.08-1.30) | 0.001 |
| **Artificially-sweetened beverages** | | | | | |
|  | Model 3 | 1 (ref) | 0.97 (0.90-1.04) | 1.15 (1.04-1.27) | 0.045 |
|  | Model 3 (P) | 1 (ref) | 0.97 (0.90-1.04) | 1.15 (1.04-1.26) | 0.002 |
| **Pure fruit/vegetable juices** | | | | | |
|  | Model 3 | 1 (ref) | 0.90 (0.85-0.94) | 0.90 (0.81-0.99) | <0.001 |
|  | Model 3 (P) | 1 (ref) | 0.89 (0.85-0.94) | 0.89 (0.80-0.99) | <0.001 |

CMM cardiometabolic multimorbidity; HR hazard ratio; CI confidence interval; ref reference

Model 3: adjusted for age, sex, ethnicity, deprivation index, smoking status, alcohol consumption, physical activity (summed MET-h/week), sedentary time, body mass index, total sugar intake, energy intake, fat intake, vegetable and fruits intake, fish intake, red meat intake, insulin use, antihypertensive drugs use, lipid-lowering drugs use, and aspirin use

Model 3(P): adjusted for variables in model 3 and using different approach to assess the physical activity by dividing participants into two groups based on whether they met the 2017 UK Physical activity guidelines of 150 minutes of walking or moderate activity per week or 75 minutes of vigorous activity
