# Supplementary material for: Associations between consumption of three types of beverages and risk of cardiometabolic multimorbidity in UK Biobank participants: a prospective cohort study
Source: BMC Med. 2022 Aug 18;20:273. doi: 10.1186/s12916-022-02456-4 (PMC9386995; doi:10.1186/s12916-022-02456-4)
Supplement: Supplementary file 9 — Additional file 9: Table S8. CMM risks with or without adjustments for body mass index in UK Biobank at 2021 (N=37,994). To investigate whether the effects of three types of beverages’ consumption were partially mediated via obesity, we re-ran the models without adjusting for BMI. CMM cardiometabolic multimorbidity, BMI body mass index (DOCX 19 kb) [file 12916_2022_2456_MOESM9_ESM.docx]

**Table S8 CMM risks with or without adjustments for body mass index in UK Biobank at 2021 (N=37,994)**

|  | | **0/day**  **HR (95% CI)** | **0-1/day**  **HR (95% CI)** | **>1/day**  **HR (95% CI)** | ***P* value**  **for trend** |
| --- | --- | --- | --- | --- | --- |
| **Sugar-sweetened beverages** | | | | | |
|  | Model 3 | 1 (ref) | 1.01 (0.95-1.07) | 1.19 (1.08-1.31) | 0.005 |
|  | Model 3 (B) | 1 (ref) | 1.02 (0.96-1.08) | 1.25 (1.13-1.37) | <0.001 |
| **Artificially-sweetened beverages** | | | | | |
|  | Model 3 | 1 (ref) | 0.97 (0.90-1.04) | 1.15 (1.04-1.27) | 0.045 |
|  | Model 3 (B) | 1 (ref) | 1.03 (0.96-1.10) | 1.31 (1.18-1.44) | <0.001 |
| **Pure fruit/vegetable juices** | | | | | |
|  | Model 3 | 1 (ref) | 0.90 (0.85-0.94) | 0.90 (0.81-0.99) | <0.001 |
|  | Model 3 (B) | 1 (ref) | 0.88 (0.84-0.93) | 0.89 (0.81-0.99) | <0.001 |

CMM cardiometabolic multimorbidity; HR hazard ratio; CI confidence interval; ref reference

Model 3: adjusted for age, sex, ethnicity, deprivation index, smoking status, alcohol consumption, physical activity, sedentary time, body mass index, total sugar intake, energy intake, fat intake, vegetable and fruit intake, fish intake, red meat intake, insulin use, antihypertensive drugs use, lipid-lowering drugs use, and aspirin use

Model 3(B): adjusted for variables in model 3 without body mass index
