# Supplementary material for: Associations between consumption of three types of beverages and risk of cardiometabolic multimorbidity in UK Biobank participants: a prospective cohort study
Source: BMC Med. 2022 Aug 18;20:273. doi: 10.1186/s12916-022-02456-4 (PMC9386995; doi:10.1186/s12916-022-02456-4)
Supplement: Supplementary file 10 — Additional file 10: Table S9. CMM risks with or without mutual adjustments for other kinds of beverages’ consumption in UK Biobank at 2021 (N=37,994). To minimize the confounding effects of other beverages consumption, we re-run the analysis mutually adjusted for 3 different types of beverages, i.e., SSBs were adjusted for ASBs and pure fruit/vegetable juices intake, and vice versa. CMM cardiometabolic multimorbidity, SSBs sugar-sweetened beverages, ASBs artificially-sweetened beverages (DOCX 19 kb) [file 12916_2022_2456_MOESM10_ESM.docx]

**Table S9 CMM risks with or without mutual adjustments for other kinds of beverages’ consumption in UK Biobank at 2021 (N=37,994)**

|  | | **0/day**  **HR (95% CI)** | **0-1/day**  **HR (95% CI)** | **>1/day**  **HR (95% CI)** | ***P* value**  **for trend** |
| --- | --- | --- | --- | --- | --- |
| **Sugar-sweetened beverages** | | | | | |
|  | Model 3 | 1 (ref) | 1.01 (0.95-1.07) | 1.19 (1.08-1.31) | 0.005 |
|  | Model 3 (M) | 1 (ref) | 1.01 (0.95-1.07) | 1.17 (1.06-1.28) | 0.004 |
| **Artificially-sweetened beverages** | | | | | |
|  | Model 3 | 1 (ref) | 0.97 (0.90-1.04) | 1.15 (1.04-1.27) | 0.045 |
|  | Model 3 (M) | 1 (ref) | 0.96 (0.90-1.03) | 1.14 (1.03-1.26) | 0.032 |
| **Pure fruit/vegetable juices** | | | | | |
|  | Model 3 | 1 (ref) | 0.90 (0.85-0.94) | 0.90 (0.81-0.99) | <0.001 |
|  | Model 3 (M) | 1 (ref) | 0.89 (0.85-0.94) | 0.90 (0.80-0.99) | <0.001 |

CMM cardiometabolic multimorbidity; HR hazard ratio; CI confidence interval; ref reference

Model 3: adjusted for age, sex, ethnicity, deprivation index, smoking status, alcohol consumption, physical activity, sedentary time, body mass index, total sugar intake, energy intake, fat intake, vegetable and fruit intake, fish intake, red meat intake, insulin use, antihypertensive drugs use, lipid-lowering drugs use, and aspirin use

Model 3(M): adjusted for variables in model 3 and other kinds of sweet beverages (artificially-sweetened beverages and pure fruit/vegetable juices consumption for sugar-sweetened beverages; sugar-sweetened beverages and pure fruit/vegetable juices consumption for artificially-sweetened beverages; sugar-sweetened beverages and artificially-sweetened beverages consumption for pure fruit/vegetable juices)
