# Supplementary material for: Associations between consumption of three types of beverages and risk of cardiometabolic multimorbidity in UK Biobank participants: a prospective cohort study
Source: BMC Med. 2022 Aug 18;20:273. doi: 10.1186/s12916-022-02456-4 (PMC9386995; doi:10.1186/s12916-022-02456-4)
Supplement: Supplementary file 11 — Additional file 11: Table S10. CMM risks according to the consumption of three types of beverages using multi-state model in UK Biobank at 2021 (N=119,589). Using multi-state model to recalculate the associations between three types of beverages and cardiometabolic outcomes, which was the classical and reliable approach for multimorbidity study. The MSM approach allowed simultaneous estimation of the role of risk factors in the transitions, which included from healthy to single cardiometabolic disease and from single cardiometabolic disease to cardiometabolic multimorbidity. CMM cardiometabolic multimorbidity. [file 12916_2022_2456_MOESM11_ESM.docx]

**Table S10 CMM risks according to the consumption of three types of beverages using multi-state model in UK Biobank at 2021 (N=119,589)**

|  | | **healthy→**  **single cardiometabolic disease** | **single cardiometabolic disease→cardiometabolic multimorbidity** |
| --- | --- | --- | --- |
| **Sugar-sweetened beverages** | | | |
|  | 0/day | 1 (ref) | 1 (ref) |
|  | 0-1/day | 1.03 (0.99-1.07) | 0.93 (0.84-1.03) |
|  | >1/day | 1.25 (1.18-1.32) | 1.18 (1.02-1.36) |
| **Artificially-sweetened beverages** | | | |
|  | 0/day | 1 (ref) | 1 (ref) |
|  | 0-1/day | 1.06 (0.98-1.13) | 1.01 (0.93-1.10) |
|  | >1/day | 1.15 (1.07-1.26) | 1.24 (1.09-1.41) |
| **Pure fruit/vegetable juices** | | | |
|  | 0/day | 1 (ref) | 1 (ref) |
|  | 0-1/day | 0.90 (0.86-0.96) | 0.89 (0.80-0.91) |
|  | >1/day | 0.92 (0.87-0.98) | 0.88 (0.82-0.96) |

CMM cardiometabolic multimorbidity; ref reference

Adjusted for age, sex, ethnicity, deprivation index, smoking status, alcohol consumption, physical activity, sedentary time, body mass index, total sugar intake, energy intake, fat intake, and dietary pattern.

In the multi-state model, we included 144,979 participants who completed the online 24-h dietary recall questionnaire on at least one occasion and did not have any history of single cardiometabolic disease (coronary heart disease, hypertension, stroke or diabetes) or CMM at baseline. We also excluded participants who had missing data (n=19,808) or who reported dubious intake of total energy (n=5,582) as we did in the main analysis, leaving 119,589 participants in the MSM analysis. These participants were regarded as “healthy” status in our analysis. The diagnoses of cardiometabolic disease were conducted as we did in the main analysis. Participants who were diagnosed with only one cardiometabolic disease during the follow-up were regarded as “single cardiometabolic disease” status. Participants who were diagnosed with two or more cardiometabolic disease during the follow-up were regarded as “cardiometabolic multimorbidity” status.
